# Pemphigus vulgaris with exclusive manifestation in one of monozygotic twins: could environmental factors be involved?^☆^

**DOI:** 10.1016/j.abd.2021.04.017

**Published:** 2022-07-09

**Authors:** Marcela Rosa de Almeida Farid, Roberto Bueno-Filho, Eduardo Antônio Donadi, Ana Maria Roselino

**Affiliations:** Department of Internal Medicine, Hospital das Clínicas, Faculty of Medicine, Universidade de São Paulo, São Paulo, SP, Brazil

Dear Editor,

Pemphigus vulgaris (PV) affects mainly the mucous membranes, through the production of autoantibodies against desmoglein (Dsg) 3. Anti-Dsg1 and anti-Dsg3 autoantibodies are produced in the mucocutaneous form of the disease. The incidence of PV has been increasing in the northeastern region of the state of São Paulo, Brazil, an endemic region for pemphigus foliaceus (PF),^1^ Susceptible/protective HLA alleles for PV,^2^ agricultural activities, and, more recently, salivary proteins from insect bites^3^ have been described in association with PV.

Reports of monozygotic twins affected by PV are rare^4^, ^5^ (Table 1). The present report describes the third case of monozygotic twin sisters, 43-years-old, of which only one developed PV. In September 2018, the twin with PV had multiple oral erosions (Fig. 1a). Direct Immunofluorescence (DIF) on the Tzanck smear showed fluorescence with anti-IgG on the keratinocytes cell membrane (Fig. 1b); the oral mucosal biopsy demonstrated acantholytic suprabasal bullae (Fig. 1c). Multinucleated keratinocytes suggestive of HSV infection were also observed in the Tzanck smear. DIF on the skin biopsy and indirect immunofluorescence (IIF) with normal skin substrate presented fluorescence with anti-IgG on the keratinocytes cell membrane. Prednisone 40 mg/day, dapsone 50 mg/day, and folic acid 5 mg/day were prescribed. After a few days, lesions appeared on the abdomen, anterior chest region, and back (Fig. 1d), with the presence of Nikolsky sign. Biopsy of the lesion on the back showed the presence of an acantholytic suprabasal bulla on histopathology, and interkeratinocyte fluorescence for IgG and C3 on DIF. After eight months of treatment, dapsone was replaced by cyclophosphamide at 50 mg/day. In March 2020, the patient suspended the medication due to the coronavirus pandemic. In June 2020, the oral lesions recurred and prednisone was restarted.

**Table 1:** Clinical and laboratory data obtained from the literature on monozygotic twins presenting with pemphigus vulgaris.

Reference		Clinical form of pemphigus vulgaris	Sex	Age (years)	IIF	Anti-Dsg1 (U/mL)	Anti-Dsg3 (U/mL)	HLA Alleles/haplotypes^c^
Ruocco et al. (1985)^4^ Italy	Twin 1	Mucocutaneous	Female	11½	1:40	ND	ND	A*24, A*29; B*45, B*35; C*04, C*x; DRB1*04, DRB1*07
	Twin 2	Not affected^a^		NA	1:20	ND	ND	
Salathiel et al. (2016)^5^	Twin 1	Mucocutaneous	Female	11	IgG	120.77	160.82	DRB1*04:02-DQA1*03:01-DQB1*03:02
Brazil	Twin 2			16	IgG	74.02	151.66	DRB1*14:04-DQA1*01:01-DQB1*05:03
Farid et al. (2021)	Twin 1	Mucocutaneous	Female	43	IgG	2.0±	189.1	DRB1*08:04, DRB1*14:01; DQA1*01:01, DQA1*05:01; DQB1*03:01^+^, DQB1*05:01^++^
Brazil (current)	Twin 2	Not affected^b^		NA	IgG	1.6	0.7	

IIF, Indirect immunofluorescence; Dsg, Desmoglein; ND, Not determined; NA, Not affected.

a At 23 years old.

b At 45 years old; ± anti-Dsg1 titer when the patient had exclusive oral lesions.

c PV susceptibility alleles are underlined; resistance + and susceptibility++ alleles to pemphigus foliaceus (see Ref. ^2^.

**Figure 1:**
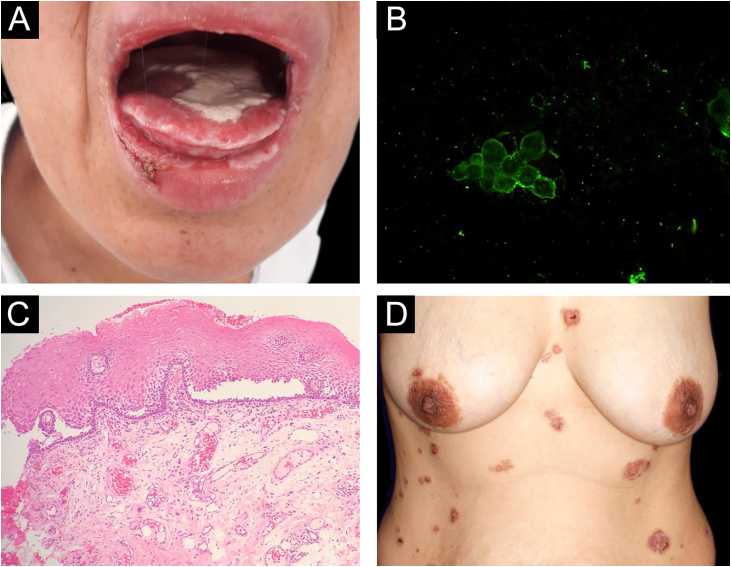
(A), Lesions on the oral mucosa: erosions on the tongue covered by a whitish pseudomembrane, erosions on the labial mucosa, erosions and crusts on the lower lip. (B), Direct immunofluorescence on oral Tzanck smear cytology: fluorescence with anti-IgG on the keratinocytes cell membrane. (C), Histopathology of the oral mucosa (Hematoxylin & eosin, ×40): acantholytic suprabasal bulla. (D), Skin lesions: erythematous plaques on the abdomen and chest with bullae, erosions and hematic crusts.

When the patient had exclusive mucosal lesions, the ELISA test resulted in anti-Dsg1 2.0 U/mL and anti-Dsg3 189.1 U/mL (MBL, Japan, cutoff of 20 U/mL for both), while her sister showed negative values ​​on ELISA (1.6 U/mL and 0.7 U/mL, respectively) and IIF.

Both sisters had two HLA susceptibility alleles for PV: HLA-DRB1*08:04 and HLA-DRB1*14:01, while the *HLA-DQA1* alleles were not associated with PV.^2^ Moreover, they had two other alleles, HLA-DQB1*03:01, and HLA-DQB1*05:01, which have been described in association with resistance and susceptibility to PF, respectively^2^ (Table 1).

Two years later, only one of the twins had PV. Although they had an identical HLA profile, they had not been exposed to similar environmental factors. Both lived in a region prevalent for PV,^1^ in the northeastern region of the state of São Paulo, Brazil. However, the affected sister had worked in rural activities, on a chicken farm, for 10 years, while her unaffected twin sister reported having always worked in the urban area. Rural workers are more exposed to pesticides, as well as to another possible PV trigger – blood-sucking insect bites.^3^

When evaluating the HLA profile and environmental factors in PV triggering, one can detect, in advance, those individuals who are most likely to develop the disease. And the question arises whether by modifying the contributing factors, whenever possible, one could prevent the emergence of PV.

In conclusion, the genotype-environmental factor interactions need to be further explored in the pathogenesis of pemphigus, and the present report illustrates this scenario.

## Financial support

This work was partially supported by FAPESP (*Fundação de Amparo à Pesquisa do Estado de São Paulo*; #2010/51729-2) and FAEPA (*Fundação de Apoio ao Ensino, Pesquisa e Assistência*).

## Authors' contributions

Marcela Rosa de Almeida Farid: Approval of the final version of the manuscript; design and planning of the study; drafting and editing of the manuscript; collection, analysis, and interpretation of data; critical review of the literature.

Roberto Bueno-Filho: Approval of the final version of the manuscript; collection, analysis, and interpretation of data; intellectual participation in the propaedeutic and/or therapeutic conduct of the studied cases; critical review of the manuscript.

Eduardo Antônio Donadi: Approval of the final version of the manuscript; collection, analysis, and interpretation of data; critical review of the manuscript.

Ana Maria Roselino: Approval of the final version of the manuscript; design and planning of the study; drafting and editing of the manuscript; collection, analysis, and interpretation of data; effective participation in research orientation; intellectual participation in the propaedeutic and/or therapeutic conduct of the studied cases; critical review of the literature; critical review of the manuscript.

## Conflicts of interest

None declared.
